# Visualization method for stress-field evolution during rapid crack propagation using 3D printing and photoelastic testing techniques

**DOI:** 10.1038/s41598-018-22773-0

**Published:** 2018-03-12

**Authors:** Yang Ju, Heping Xie, Xi Zhao, Lingtao Mao, Zhangyu Ren, Jiangtao Zheng, Fu-Pen Chiang, Yongliang Wang, Feng Gao

**Affiliations:** 1 State Key Laboratory of Coal Resources and Safe Mining, China University of Mining & Technology, Beijing, 100083 China; 2 State Key Laboratory for Geomechanics and Deep Underground Engineering, China University of Mining & Technology, Xuzhou, 221116 China; 3 Institute of Deep Earth Science and Green Energy, Shenzhen University, Shenzhen, 518060 China; 4 School of Mechanics & Civil Engineering, China University of Mining & Technology, Beijing, 100083 China; 5 Department of Mechanical Engineering, Stony Brook University, Stony Brook, NY 11794-2300 USA

## Abstract

Quantitative visualization and characterization of stress-field evolution during fracture rapid growth is critical for understanding the mechanisms that govern the deformation and failure of solids in various engineering applications. However, the direct capture and accurate characterization of a rapidly-changing stress field during crack propagation remains a challenge. We report an experimental method to quantitatively visualize and characterize rapid evolution of the stress-field during crack propagation in a transparent disc model containing a penetrating fusiform crack. Three-dimensional (3D) printing technology and a stress-sensitive photopolymer resin were adopted to produce the disc model and to alleviate the residual processing stress that usually blurs the dynamic stress field due to overlap. A photoelastic testing system that synchronized a high-speed digital camera and a pulsed laser with a nanosecond full width at half maximum (FWHM) was used to capture the rapid evolution of the stress field in the vicinity of crack tips. The results show that the proposed method is suitable to directly visualize and quantitatively characterize the stress-field evolution during crack rapid propagation. It is proved that the crack propagation velocity is strongly governed by the stress field around the crack tips.

## Introduction

Load-induced stress fields govern the deformation and fracture behaviour of solids. Knowledge of how those stress fields evolve yields valuable information that is used in a wide variety of practical applications^1–13^. In geoscience, for instance, laboratory observations of stress wave and crack propagation provide valuable information on the rupturing process of earthquake faults^2,3^. In machinery manufacturing, the strength and fatigue-life prediction of a component depends on its geometry and stress state^4–6^. In medical science, the mechanical resistance of fractured bone fixations in surgery acts as an input for clinical decision-making related to fixation methods^7^. Underground engineering, roadway excavation^8^, surrounding rock stability^9,10^, stress-controlled fluid flow in geological disposal of nuclear waste^11^ and hydraulic-driven oil and gas exploitation^12,13^ all involve, to varying degrees, the dynamic stress redistribution in rock masses induced by rock fracture.

Over the past decades, a large number of theoretical models^1,14–18^ and numerical methods^19–23^ have been proposed to characterise the rapid evolution of stress-field in solid materials. However, because of the simplification of models’ geometries and boundary conditions, the analytical results of the complex fractures have sparked significant debate owing to the lack of direct experimental validation^24–26^. The direct capture and experimental visualization of the dynamic stress evolution during fracture rapid propagation remains challenging^26,27^.

In pioneering studies that aimed to experimentally probe the mechanical properties and fracture behaviour of solids, photoelastic tests combined with a high-speed camera were used to observe the isochromatic fringe patterns during fracturing^1,24,26–39^. For instance, Bieniawski^1^ obtained a photographic record of propagating rock fracture using birefringent coating technique combined with a high-speed camera and explored the stability of the fracture propagation, terminal fracture-velocity and dynamic stresses generated by the propagating crack. Using marble specimens coasted with brittle polyester, Daniel and Rowlands^24^ observed isochromatic fringe patterns during crack propagation loaded by a steel wedge in a notch, however, the results obtained were only qualitative. Dally^30^ studied dynamic-fracture characteristics of the metallic materials by using birefringent coating techniques and reviewed methods for determining the stress intensity factor K(t) from these patterns. Xia *et al*. observed a shear crack propagating between two frictionally held or weakly boned identical pieces of brittle polyester resin under far-field asymmetric loading and found that the shear crack propagated faster than the shear wave speed^2,3,33^. They also investigated spontaneous mixed-mode fractures and self-similar crack growth behaviour^34–36^. Gomez *et al*.^37^ captured the photoelastic fringe patterns of a Homalite-100 disc under dynamic tensile splitting and analysed the damage regions. All of these studies offer straightforward approaches for capturing the dynamic-stress status in the fracture of a solid material yet lack quantitative visualisation of the continuous evolution of the stress field during crack rapid propagation.

The application of a transparent brittle polyester and photoelastic coating^28–31,38^ makes it possible to examine the dynamic fracture characteristics of opaque solid materials. However, the coating technique requires elaborate surface preparation of both the specimen and the coating itself and the results suffered from uncertainty because the coating failure responds to fracture in the sample had not been thoroughly discussed and a big limitation is that the results only show us what happens at the surfaces, not within the opaque materials^26,27^. In addition, the residual stress resulting from coating processes influences the accuracy of stress status of the sample. Therefore, the most obvious barriers to investigating the essential mechanism that governs the fast fracture growth and failure of a brittle material are the lack of an accurate, quantitative characterization of rapidly evolving stress fields during fracturing and the relationship between near-tip stress and fracture features^26,27,39^. A valid quantitative visualisation of the instantaneous stress field that relates crack propagation in a solid material to its birefringence and elastic-brittle fracture properties is needed.

To achieve this goal, in this study, we synchronized a photoelastic testing equipment and a high-speed photographic system to instantaneously capture a sequence of isochromatic fringe patterns in a transparent disc specimen during diametrical splitting. To facilitate preparing the specimen and to avoid the residual stress that is usually associated with a traditional modelling process, 3D printing technology and a stress-sensitive photopolymer resin which exhibited elastic brittle behaviour during tension were used to fabricate a transparent seamless disc specimen containing a penetrating fusiform crack. A 220-nanosecond FWHM pulsed laser source was synchronized with the shooting frequency of the high-speed camera. The stress evolution was qualitatively characterized as well as the stress intensity factor in rupture. The numerical results of the dynamic stress field incorporating linear elastic fracture mechanics (LEFM) were presented for the sake of comparison.

## Materials and Methods

### Fusiform crack discs

**Figure 1:**
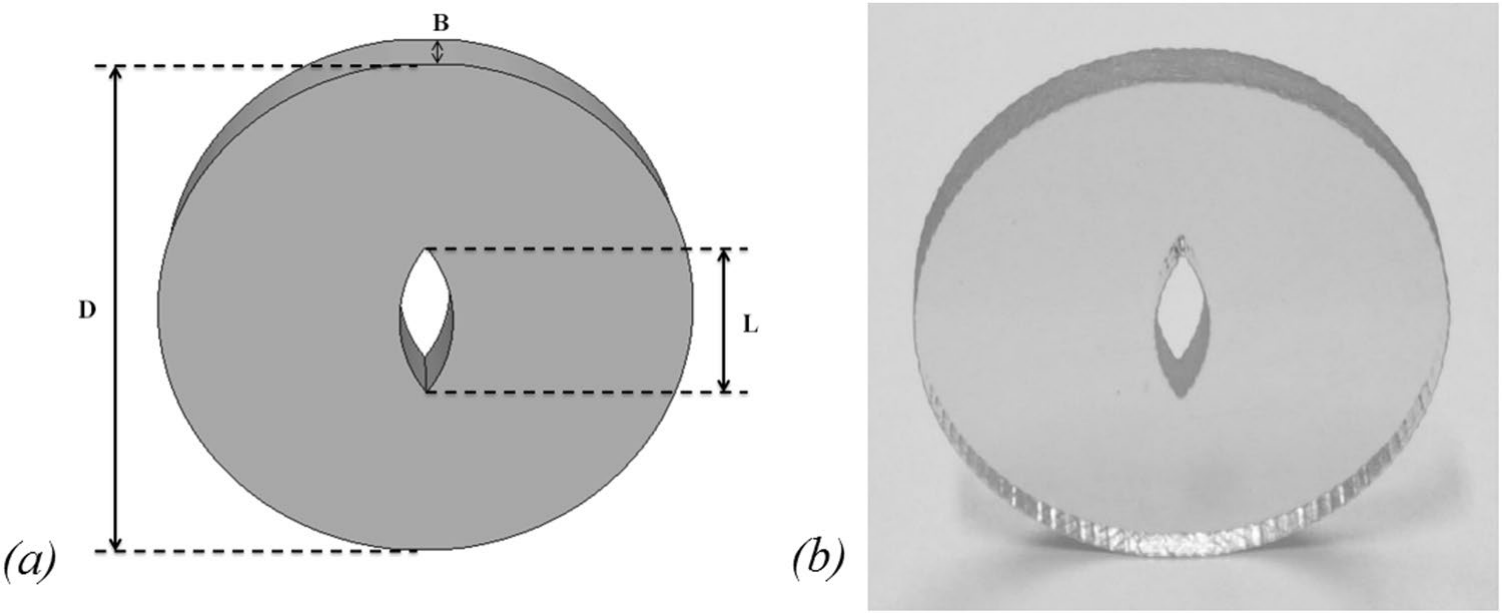
Fusiform crack disc. (**a**) Schematic of the disc; diameter *D* = 50 mm, thickness *B* = 7.76 mm and internal aperture length *L* = 15 mm; (**b**) photograph of printed photosensitive resin sample.

A 3D printing technique was used to manufacture the integrated seamless, transparent disc containing a penetrating fusiform crack (shown in Fig. 1). (See the Supplementary Information (SI) for details). 3D printing technique is an emerging 3D prototyping or printing technology which realizes a rapid manufacturing of a complicated 3D solid. It adopts the digital files of a target body. The 3D body is formed by spraying powder (or liquid) photopolymer, ceramic, or even metal, layer by layer, before being cured by laser^40,41^. In contrast to the previous investigations^33^ that adopted a bonded disc model using two identical pieces of polyester resin with an in-built weak interface that directed crack propagation, our study produced an integrated model without preset weak interfaces, focusing on the dynamic evolution of the stress field that associates with crack initiation and propagation.

**Figure 2:**
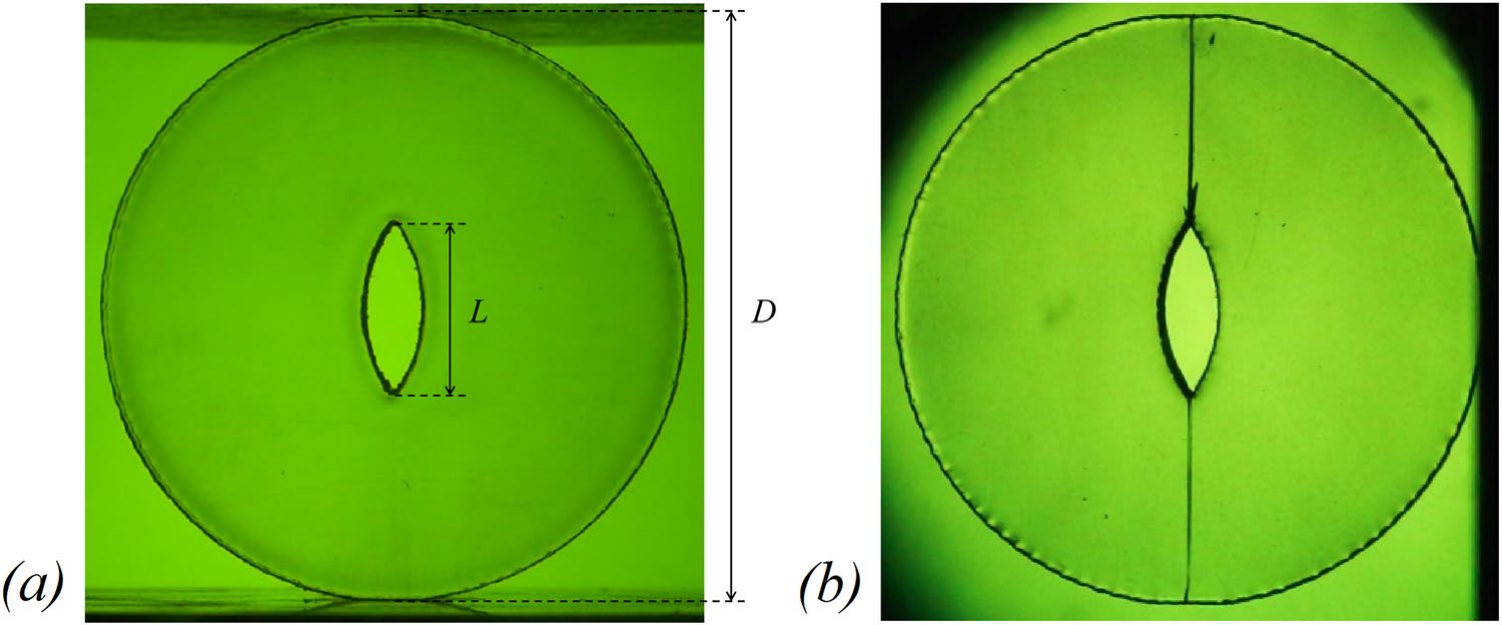
Disc (**a**) before and (**b**) after loading in a circularly-polarized field, when the crack appears (*D* = 50 mm, *L* = 15 mm).

Our fusiform-crack disc has components and photoelastic properties similar to the traditional photoelastic material epoxy resin^42,43^. It is a kind of transparent polyester material and has birefringent effect under loading, which makes it possible, from the theoretical point of view, to apply 3D printed disc to study the stress distribution. In addition, the disc before and after loading in a circularly-polarized field (details see section 2.2) has no isochromatic fringe, which indicates minimal residual stress due to the manufacturing process left in the model (illustrated in Fig. 2a) and failure behaviour similar to brittle material with no apparent residual stress (Fig. 2b). Its elastic failure behaviour ensures the application of the photoelastic test and theory to observe the dynamic isochromatic fringe and quantitatively analyse the stress field evolution.

### Fracture recording

**Figure 3:**
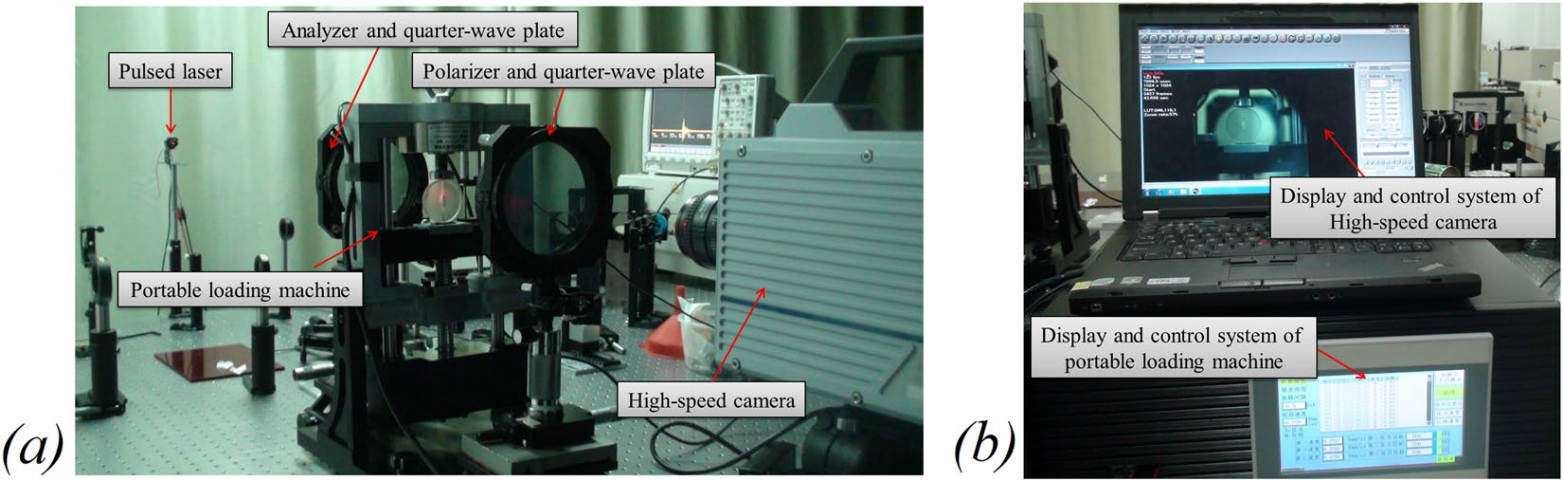
High-speed photoelastic testing system. (**a**) Pulsed-laser light source, portable loading machine, polarizer, wave plate and high-speed camera; (**b**) display and control systems of the high-speed camera and portable loading machine

The high-speed photoelastic system consisted of a pulsed laser-light source, optical instruments (including two polarizers and two quarter-wave plates), a high-speed camera (5,000–650,000 frames per second) and a portable motorized loading machine. Figure 3 shows a schematic of the setup, during the recording process, collimated laser light passes through the expander, the polarizer, the first quarter-wave plate, the specimen under diametric compression by portable loading machine, the second quarter-wave plate and the analyser and finally imaging interferometric as isochromatic fringes in the charge-coupled device of the high-speed camera.

**Figure 4:**
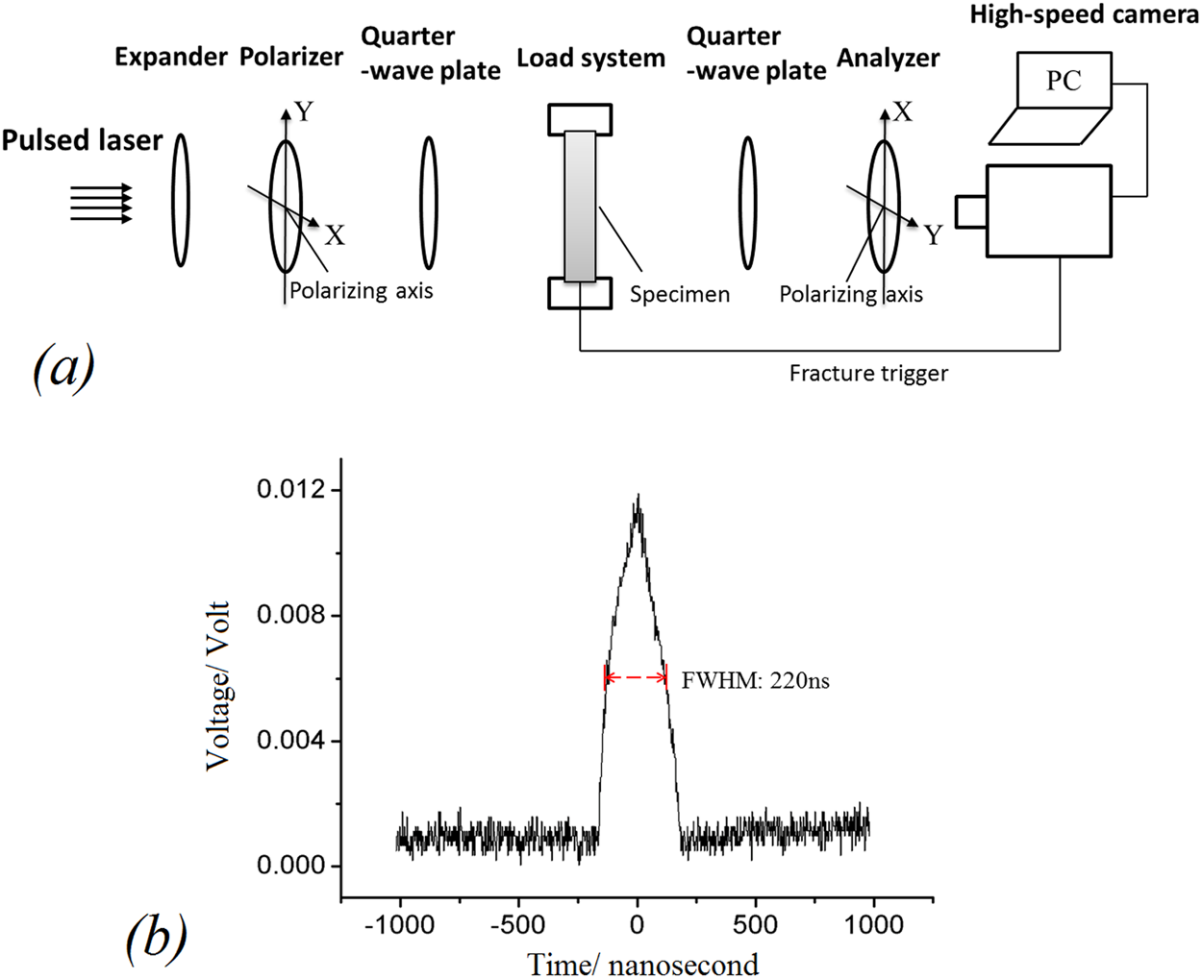
(**a**) The optical arrangement that produces the circularly-polarized field; (**b**) oscillogram and characters of the nanosecond FWHM pulsed-laser source.

**Figure 5:**
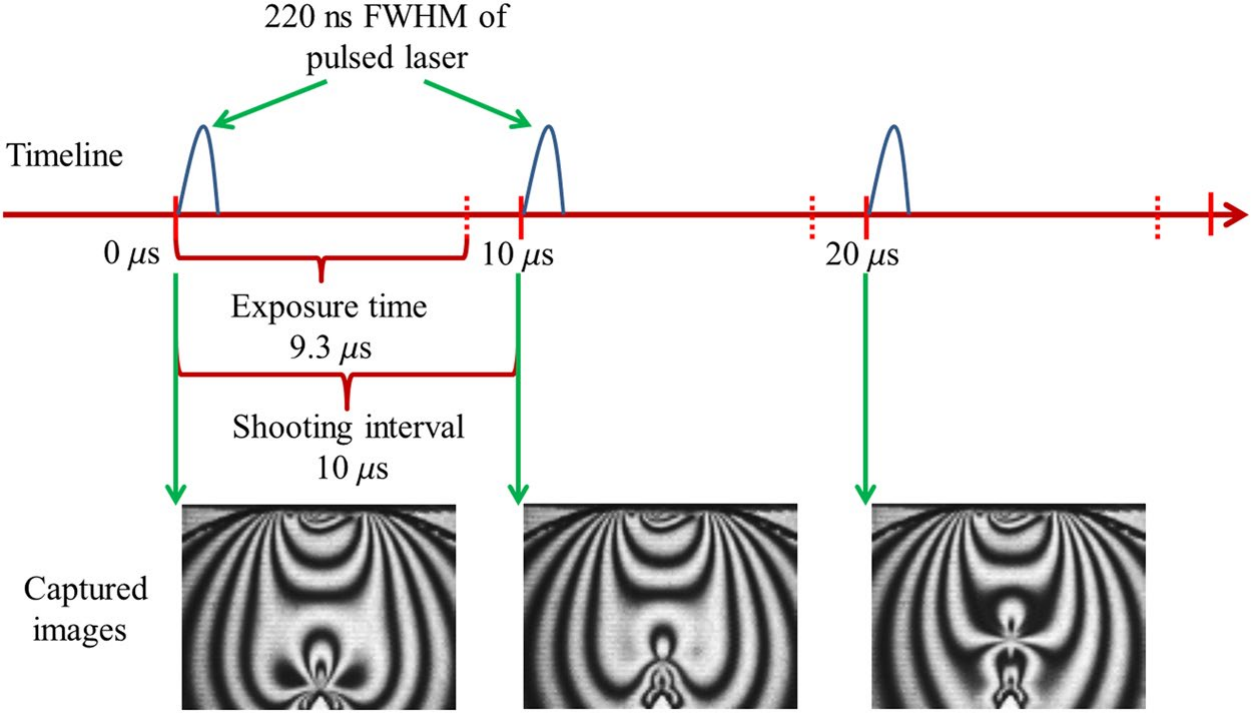
Diagram of the pulsed laser source synchronized with the photo-frequency of the high-speed digital camera.

The optical arrangement that produces the circularly-polarized field is illustrated in Fig. 4a,b shows the oscillogram of the pulsed-laser source. To capture high-quality dynamic isochromatic fringes, pulsed laser light source replaces the continuous light and we set the pulsed laser source frequency to 100 KHz, to match the 100,000 frames per second captured by our high-speed camera, and a FWHM of 220 ns, which is 1/42 of the photographic exposure time of 9.3 µs. Figure 5 show the fringe patterns captured using pulsed laser. They demonstrate that high-quality dynamic isochromatic patterns can be clearly captured by the pulsed laser instead of continuous light (results using continuous light can be seen in SI) and the pulsed laser source is sufficiently small to ensure that the images captured are sharp.

### Analysis approach

The time-lapse images of isochromatic fringes were converted into deviator stresses through plane photoelasticity^44^ to quantify the dynamic stress field during fracturing. In 3D stress field, any point under loading can be expressed to 3D stress state by six stress components including *σ*_*x*_, *σ*_*y*_, *σ*_*z*_, *σ*_*z*_, *τ*_*xy*_, *τ*_*yz*_ and *τ*_*zx*_ or three principal stresses which are *σ*_1_, *σ*_2_ and *σ*_3_. While in plane stress state (*x*-*y* plane), the stress components in *z* direction equal to zero (*σ*_*z*_ = *τ*_*zx*_ = *τ*_*zy*_), then the stress state of any point can be expressed by three stress components (*σ*_*x*_, *σ*_*y*_ and *τ*_*xy*_) or two principal stresses *σ*_1_ and *σ*_2_. And their relationship can be expressed as:

1 $${\sigma }_{1}-{\sigma }_{2}=\sqrt{{({\sigma }_{x}-{\sigma }_{y})}^{2}+4{\tau }_{xy}^{2}}$$

In the circularly-polarized field, isochromatic fringes appear continuously; to analyse the stress field, the fringes need to first be separated. The circularly-polarized field is set as the bright field, and from the free boundary to the crack tip the isochromatic fringe order *n* gradually increases by a half-integer. In the stress-optical law^45^, incident light decomposes into two plane polarized lights along the directions of two plane principal stresses, with optical path difference *dδ*; optical path difference is associated with a series of optical, stress coefficient:

2 $$d\delta =\lambda /{f}_{0}({\sigma \text{'}}_{1}-{\sigma \text{'}}_{2})dz$$

where *λ* is incident light wavelength, *f*_0_ represents the fringe constant of the photosensitive resin, *d*z the thickness of the plane, ${\sigma \text{'}}_{1}$ and ${\sigma \text{'}}_{2}$ are principal stresses.

To simply expressing the relationship between the stresses and optical quantity, in plane stress condition, the stress intensity can be expressed as by isochromatic fringe order *n*, fringe value *f*_0_ and plane thickness *t*.

3 $${\sigma }_{1}-{\sigma }_{2}=\frac{n\cdot {f}_{0}}{t}$$

**Figure 6:**
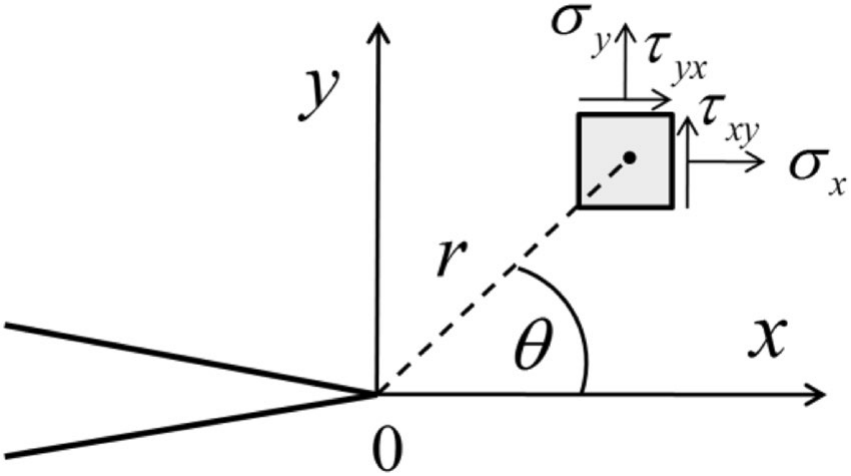
Stress components around the crack tip in the stress plane.

Following on from these theories, for plane stress, the stress components can be expressed by the Westergaard stress function^46^:

4 $$\begin{array}{c}{\sigma }_{x}=\frac{{K}_{I}}{\sqrt{2\pi r}}\,\cos \,\frac{\theta }{2}(1-\,\sin \,\frac{\theta }{2}\,\sin \,\frac{3\theta }{2})+{\sigma }_{ox}\\ {\sigma }_{y}=\frac{{K}_{I}}{\sqrt{2\pi r}}\,\cos \,\frac{\theta }{2}(1+\,\sin \,\frac{\theta }{2}\,\sin \,\frac{3\theta }{2})+{\sigma }_{oy},\\ {\tau }_{xy}=\frac{{K}_{I}}{\sqrt{2\pi r}}\,\cos \,\frac{\theta }{2}\,\sin \,\frac{\theta }{2}\,\cos \,\frac{3\theta }{2}+{\tau }_{oxy}\end{array}$$

where *x* is the crack propagation direction, *y* is the normal direction of the crack plane and *r* and *θ* are the polar coordinates (as shown in Fig. 6), *σ*_*ox*_, *σ*_*oy*_, and *τ*_*oxy*_ are the higher- order terms. Note that the distance between the point of interest whose stress distribution was determined using the Westergaard stress function and the crack tip should not be too small or too big in accordance with the requirements of Westergaard stress function^47^. It is suitable for the zone studied in our experiment.

The relationship between polar coordinates and rectangular coordinates can be expressed as

5 $$r=\sqrt{{x}^{2}+{y}^{2}},\theta =\arctan \frac{y}{x}$$

Based on Equations (1) and (3), the relationship between the isochromatic fringe and plane stress can be expressed as

6 $$\frac{n\cdot {f}_{0}}{t}=\sqrt{{({\sigma }_{x}-{\sigma }_{y})}^{2}+4{{\tau }_{xy}}^{2}}$$

Finally, through Equations (4) and (6) the stress intensity factor at the crack tip can be calculated by the isochromatic fringe order *n* and its polar coordinates *r* and *θ* (for full details, see the supplementary information):

7 $$\begin{array}{cc} & \sqrt{{(\frac{-2{K}_{I}}{\sqrt{2\pi r}}\cos \frac{\theta }{2}\sin \frac{\theta }{2}\sin \frac{3\theta }{2}+{\sigma }_{ox}-{\sigma }_{oy})}^{2}+4{(\frac{{K}_{I}}{\sqrt{2\pi r}}\cos \frac{\theta }{2}\sin \frac{\theta }{2}\cos \frac{3\theta }{2}+{\tau }_{oxy})}^{2}}\\ = & \frac{n\cdot {f}_{0}}{t}\end{array},$$

where *K*_*I*_ is the mode-I stress intensity factor, *r* and *θ* are the polar coordination components in the stress plane and *σ*_*ox*_, *σ*_*oy*_ and *τ*_*oxy*_ are the constant stress components in the stress plane.

### Linear elastic fracture mechanics (LEFM) simulation

LEFM modelling provides a theoretical framework for numerically analyzing the stress field at the near-tip region during crack propagation^48^. A comparison numerical LEFM simulation was carried out in order to validate the stress field measured. The basic assumption for the material is linear elastic, isotropic and homogeneous medium, regardless of complex energy dissipation and nonlinear problem in the fracture process. The LEFM assumes that (1) the brittle elastic fracture occurs in the close vicinity of the crack tip and (2) that a sufficiently small region of fracture develops around the tip. This approach has been used to describe the propagation of a single straight crack during brittle failure^49^, however, it is unable to represent complex dissipative and nonlinear processes involved in fracture. The use of LEFM modelling for theoretical validation and the stress-optic law for experimental interpretation, both based on elastic mechanics, ensures the effectiveness of stress field comparisons (for details of how initial crack inserting and evolution of stress field in fracturing, see the supplementary information).

## Results and Analysis

**Figure 7:**
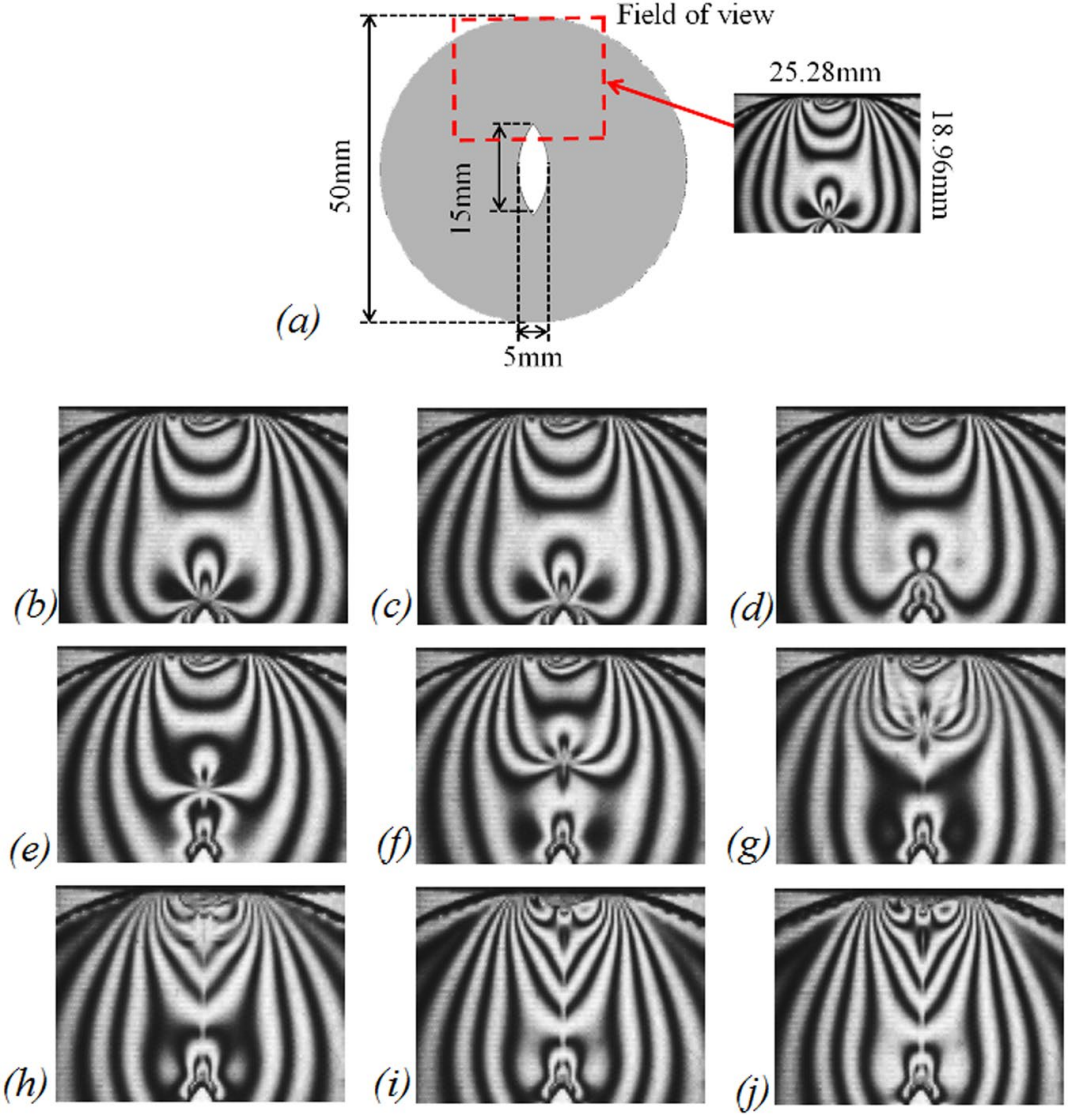
Half-integer isochromatic fringes at the near-tip region during crack propagation recorded in 10 µs intervals. (**a**) Field of view, 25.3 mm × 19.0 mm; (**b**) recorded in 0 µs; (**c**) recorded in 10 µ µs; (**d**) recorded in 20 µs; (**e**) recorded in 30 µs; (f) recorded in 40 µs; (**g**) recorded in 50 µs; (**h**) recorded in 60 µs; (**i**) recorded in 70 µs; (**j**) recorded in 80 µs.

In order to capture the dynamic isochromatic fringes in the brittle fracture zone, the field of view (25.3 mm × 19.0 mm) is located near the tip along the crack propagation path (as shown in Fig. 7a). Figure 7b–j plot the half-integer isochromatic fringes during crack propagation with framing rate of 100,000 fps. The repetitive test yield an average crack propagation speed of 275.4 m/s, ranging from 240 m/s to 320 m/s, providing us an evidence how the stress field influences the crack velocity (the repetitive test adopts the same 3D printed model with consistent material properties, geometry and loading condition; and repetitive result is provided in the supplementary information). The use of a pulsed laser light source as opposed to a continuous one enables the dynamic capture of clear isochromatic fringes because it has a frequency synchronized with that of the high-speed camera and a FWHM on the order of nanoseconds.

**Figure 8:**
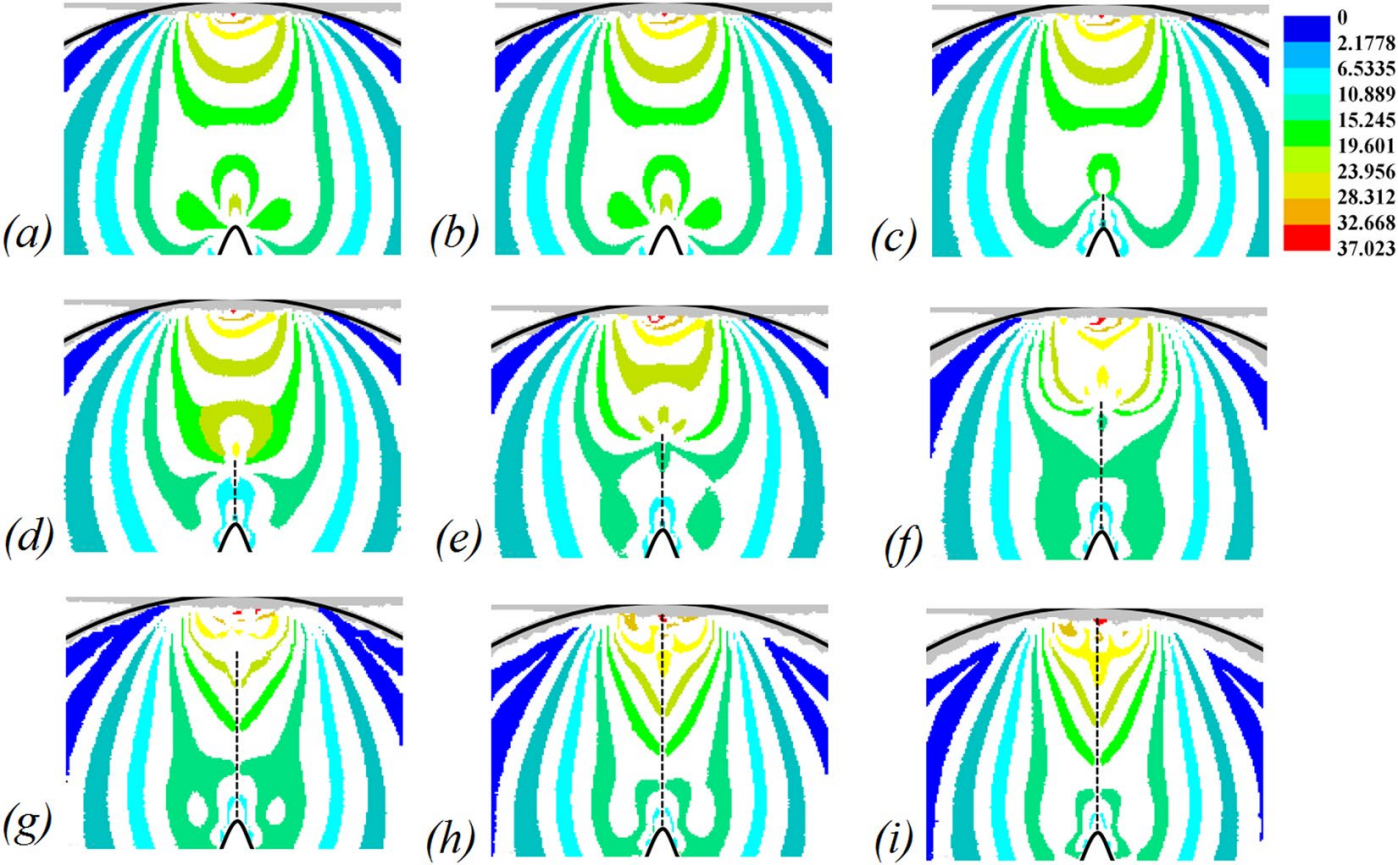
Deviator stresses at half-integer isochromatic fringes, given in MPa. (**a**) 0 µs; (**b**) 10 µs; (**c**) 20 µs; (**d**) 30 µs; (**e**) 40 µs; (**f**) 50 µs; (**g**) 60 µs; (**h**) 70 µs; (**i**) 80 µs.

The calculated deviator stresses (*σ*_1_ − *σ*_2_) at half-integer isochromatic fringes from 0 µs to 80 µs are shown in Fig. 8. The deviator stress represents the stress intensity in the physical model; larger the deviator stress, larger is the stress concentration. The crack progresses in a straight line, while the deviator stress changes with the crack length. The captured crack growth path and the varying deviator stresses indicate that the crack tip is in a high-stress state subject to diametrical compression splitting. Similar results were also observed in the previous research by the caustic method^50,51^.

**Figure 9:**
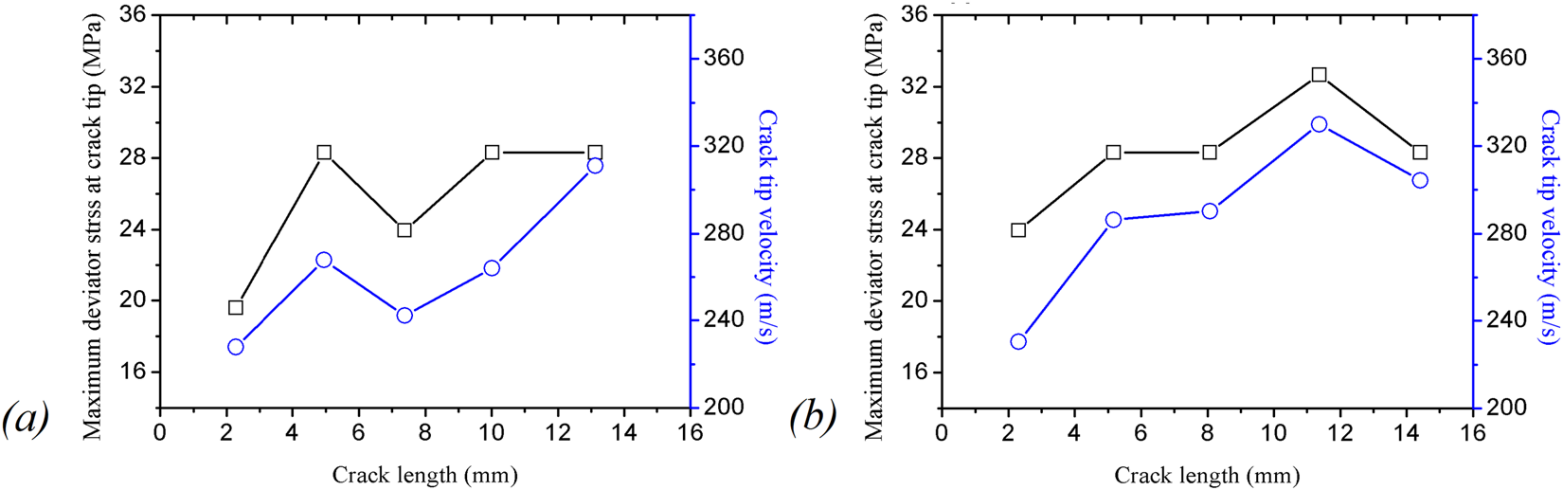
Maximum deviator stress at the crack tip and crack tip velocity as a function of crack length during crack propagation. (**a**) sample 1; (**b**) sample 2.

Figure 9 shows the maximum deviator stress at the crack tip as well as crack tip velocity as a function of crack length during crack propagation. Note that the crack propagation velocity is the average velocity within the frame interval, as shown in Fig. 5, which can be obtained by the increment of crack length within two continuous frames divided by the time between the two frames, with a shooting interval 10 µs. It can be seen that the crack-tip velocity follows the same trend as the crack-tip stress field, which means that the crack-tip stress field controls the crack velocity. To study the relationship between crack tip deviator stress *σ*_1_ − *σ*_2_, and the crack tip velocity value *ν*, a polynomial fitting function were expressed; which the fitting coefficient R-Square is 0.99634.

8 $$({\sigma }_{1}-{\sigma }_{2})=-0.00276\times {v}^{2}+1.5923\times v-199.72047$$

**Figure 10:**
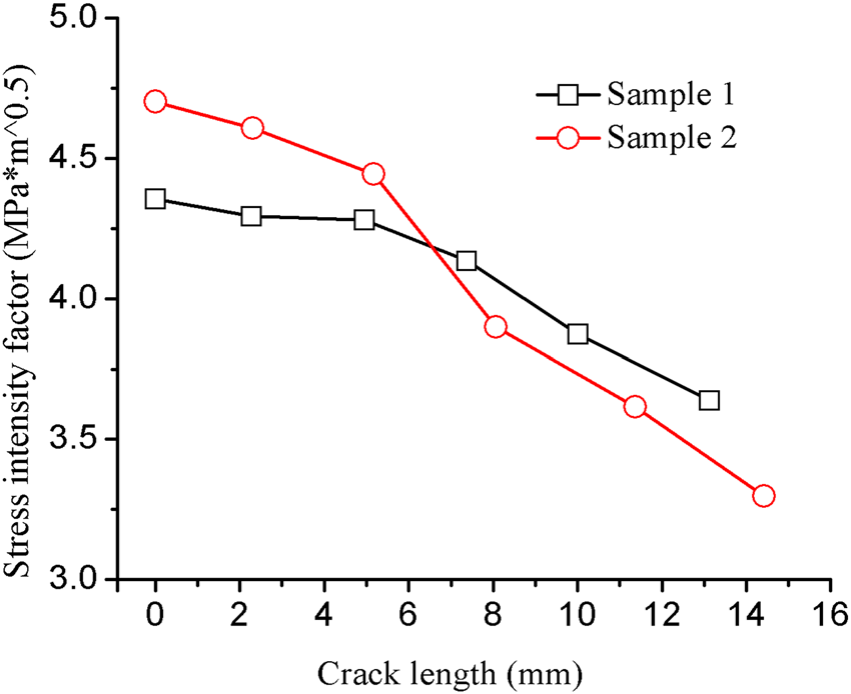
SIF during crack propagation as a function of crack length.

Next, we examined the evolution of the stress intensity factor (SIF) during crack propagation as shown in Fig. 10. We observed a decrease in SIF, which is consistent with the macroscopic physical phenomenon. The crack growth leads to the release of energy at the crack tip represented as the decrease of stress intensity factor. The comparison LEFM numerical simulation with the same model geometry, mechanical properties and loading condition (see the supplementary information) shows that the stress concentrates around the crack tip and at the upper and lower loading surfaces and moves with the crack tip, which is consistent with the recorded stress field of physical model in rupture. The accordant stress field in the simulation indicates the effectiveness of the experimental methods.

It is noteworthy that the 3D printing material that was used to fabricate specimens is not a highly brittle material, which exhibits unneglectable ductility. When the material was exposed to the continuous quasi-static load, the crack propagation speed fell into a range of 240–320 m/s, which is relatively lower than that of brittle materials under dynamic impact loads^52,53^. In principles, within the range of low crack propagation velocity (i.e., relative index v/Cs <0.3, where v is the crack propagation velocity, and Cs is the shear wave velocity), it is acceptable to use quasi-static displacement distribution for quantification of rapid evolution of the stress field around a crack tip^54^. Note that the measured shear wave velocity Cs of the printing material was 979 m/s, and the relative indices v/Cs of most measured points meet the condition v/Cs <0.3. We thus adopted the quasi-static stress field to calculate the stress intensity factor.

## Discussion

Previous analyses^16,55,56^ have suggested that the stress distribution at the crack tip changes according to the crack velocity. We know the stress field in the vicinity of a crack tip plays an important factor in understanding the behaviour of the crack propagation, however, its effect on crack velocity is not well understood. This work offers a promising way to quantify the rapid evolution of the stress field at the near-tip region during fracturing of a disc under a quasi-static compressive load. Observation of the full field of view before fracture and the arrangement of the field at the near-tip during fracturing make it possible to quantitatively understanding the relationship between the crack tip stress field and crack velocity.

Previous studies have involved elaborate surface preparation of both the specimen and the coating itself, with the coating responding to fracture in the sample and/or itself undergoing failure. The residual stress and plastic deformation in the prepared sample significantly impedes the feasibility of applying photoelastic theory. Our approach avoids the need for a photoelastic coating by using a three-dimensional printer to create a monolithic fusiform-crack disc made of photosensitive resin with birefringence and elastic-brittle fracture properties.

We observed dynamic half-integer isochromatic fringes at the near-tip region as a result of the rapid crack growth and performed a quantitative analysis of the deviator stress field near the tip. During crack growth, the crack-tip velocity and maximum deviator stress of the half-integer isochromatic fringes at the crack tip follow the same trend as a function of crack length. Our experimental result has proved the relationship between crack tip deviator stress *σ*_1_ − *σ*_2_, and the crack tip velocity value *ν*, can be expressed by a polynomial fitting function. It should be noted that the near-tip zone discussed above is not the area exactly at the crack tip or immediately close to the crack tip where the stress is infinite according of fracture mechanics theories. Instead, it is the near-tip zone where the stress is determined by the Westergaard stress function.

It appears that a full understanding of the rapidly-changing stress field in solids depends on the advanced photograph capturing system involving a pulsed light source and a high-speed camera combined with photoelasticity. It will certainly contribute to future studies of dynamic fracture behaviour in practical engineering issues, such as, in laboratory earthquake, 3D printed formation model according to the similar proportion of real formation condition can be adopted to predict to slide or rupture location and crack propagation speed which provide warning of earthquake disaster. In the aircraft industry, cracks may readily initiate from stress concentrations, such as rivet holes, and subsequently travel at velocities on the order of a few hundred meters per second. Using a similar 3D printed aircraft fuselage to physically simulate cracking process driven by explosion or inertia make it possible to redesign the geometry of aircraft and fastened rivets. In underground engineering, rock burst occurs in cavern excavation process caused by redistribution of stress field, figure out the stress field through a 3D printed cavern model is a low-cost and effective approach to do the relevant study.

## Electronic supplementary material

Supplementary Information
