# Supplementary material for: Visualization method for stress-field evolution during rapid crack propagation using 3D printing and photoelastic testing techniques
Source: Sci Rep. 2018 Mar 12;8:4353. doi: 10.1038/s41598-018-22773-0 (PMC5847554; doi:10.1038/s41598-018-22773-0)
Supplement: Supplementary file 1 — Supplementary Information [file 41598_2018_22773_MOESM1_ESM.doc]

**Supplementary information**

**Title:** Visualization method for the stress-field evolution during rapid crack propagation using 3D printing and photoelastic testing techniques

**Authors :** Yang Ju^*^, Heping Xie, Xi Zhao, Lingtao Mao, Zhangyu Ren, Jiangtao Zheng, Fu-Pen Chiang, Yongliang Wang, Feng Gao

**1 Fusiform crack disc**

A fusiform crack disc (Figure 1 main text) was manufactured using a 3D laser printer loaded with the photosensitive resin “Vero Clear” as the matrix material and the supporting material “SUP 705” as the natural crack. Vero Clear has similar photoelastic and mechanical properties to those of traditional photoelastic epoxy resin (Table S1), but it offers the advantage of rapid prototyping, easy processing, printing of complex structures and no residual stress. In addition, the cracked sample (Figure 2 main text) exhibits failure behaviour similar to a brittle material because it has no plastic zone along the tensile fracture area. Figure 1 shows a 3-mm-thick disc cut from a 50-mm-diameter and 8-mm-high cylinder; after cutting and polishing through a circularly-polarized field, no residual processing stress was found.

**Table S1** Photoelastic and mechanical properties of traditional photoelastic material (epoxy resin, polycarbonate) typically used and photosensitive resin employed in this study.

| Materials | Stress fringe values  *f_0_* (N/mm) | Elastic moduli  *E* (GPa) | Poisson's ratios  *u* |
| --- | --- | --- | --- |
| Epoxy resin | 12 | 3.3 | 0.37 |
| Polycarbonate | 8 | 2.6 | 0.28 |
| Photosensitive resin | 33.8 | 3.1 | 0.38 |


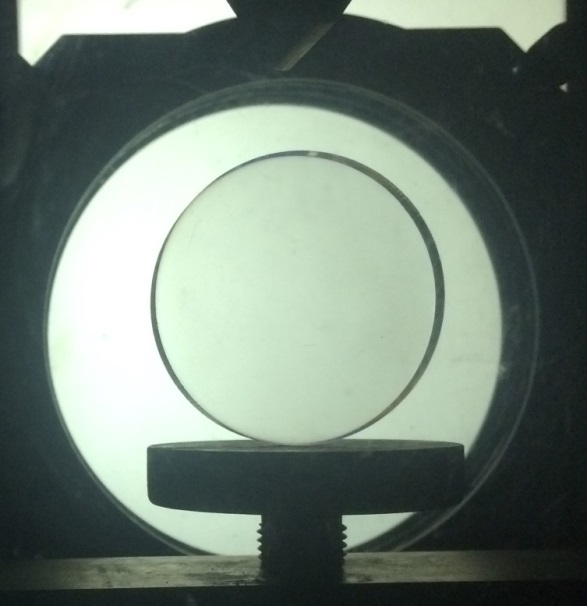


**Figure S1** Fusiform crack disc after cutting and polishing in the circularly-polarized field.

**2 Photoelastic tests**

Our high-speed photoelastic testing system consisted of optical instruments, a high-speed photography system and a portable motorized loading machine (Figure 3 in main text). In the circularly-polarized field of this system, collimated pulsed light passes through the expander, the polarizer, the first quarter-wave plate, the specimen under compression, the second quarter-wave plate and the analyser and is finally imaged as isochromatic fringes on the charge-coupled device of the high-speed camera.

When the frequency of the continuous light source does not match the shoot frequency of the high-speed camera, blurring fringes at the crack tip during crack propagation are observed. To capture high-quality dynamic isochromatic fringes, we set the pulsed light source frequency (Figure 2) to 100 KHz, to match the 100,000 frames per second captured by our high-speed camera, and a FWHM of 220 ns, which is 1/42 of the photographic exposure time of 9.3 µs. Figures 3 and 4 show the fringe patterns captured using continuous light and pulsed laser, respectively. They demonstrate that high-quality dynamic isochromatic patterns can be clearly captured by the pulsed laser instead of continuous light.


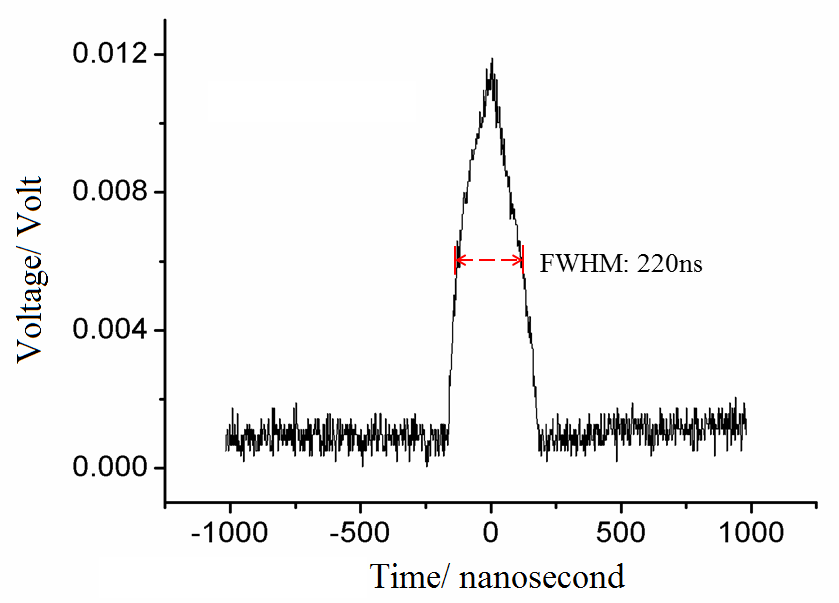


**Figure S2** The oscillogram of the pulsed light source.


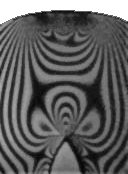

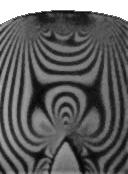

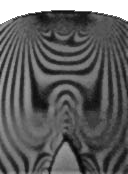

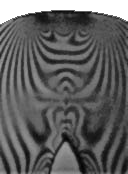


0 µs 7.4 µs 14.8 µs 22.2 µs


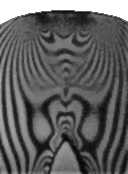

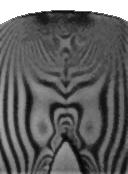

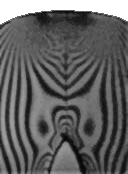

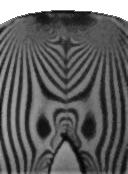


29.6 µs 37.0 µs 44.4 µs 51.9 µs

**Figure S3** Fuzzy fringe patterns observed during fracturing of the disc when using a continuous light source (captured time interval is 7.4 µs).


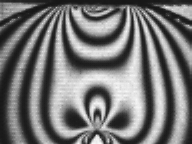

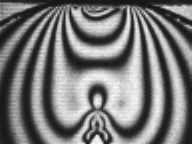

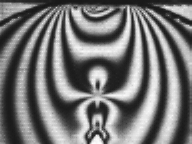


0 µs 10 µs 20 µs


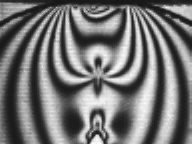

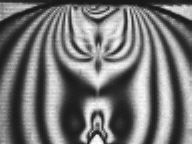

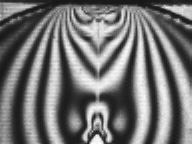


30 µs 40 µs 50 µs

**Figure S4** Sharp fringe patterns observed during fracturing of the disc using pulsed-laser light (time interval is 10 µs).

**3 LEFM simulation**

Linear elastic fracture mechanics (LEFM) [1] provides a theoretical framework for analysing the stress field at the near-tip region during crack propagation. It assumes that the material obeys linear elasticity at the very near vicinity of a crack’s tip. All of the complex dissipative and nonlinear processes that are involved in fracture are not described, and fracture is assumed to occur within a sufficiently small region around the tip. Nevertheless, this theory can be used to describe the motion of a single straight crack encountering brittle fractures [2]. LEFM also agrees with the experimental theory because the stress-optic law obeys elastic mechanics. The fracture toughness of mode I (the opening mode) is measured by the cracked chevron-notched Brazilian disc test [3] recommended by the International Society for Rock Mechanics:
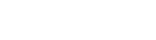
.

The geometry and boundary conditions of the numerical model are consistent with the printed specimen and experimental test. The LEFM criterion [4] was adopted to study the fracture process. Because the stress field captured in the photoelastic test system (introduced in main text) is in the stress plane state and the numerical model is in three dimensions, two equivalent principal stresses
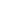
 and
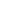
 are defined as follows [5]:


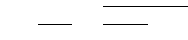
 (1)


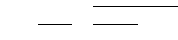
 (2)


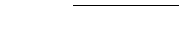
 (3)

Thus the deviator stresses in the numerical model and in the photoelastic test physical model both can be expressed as
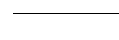
.


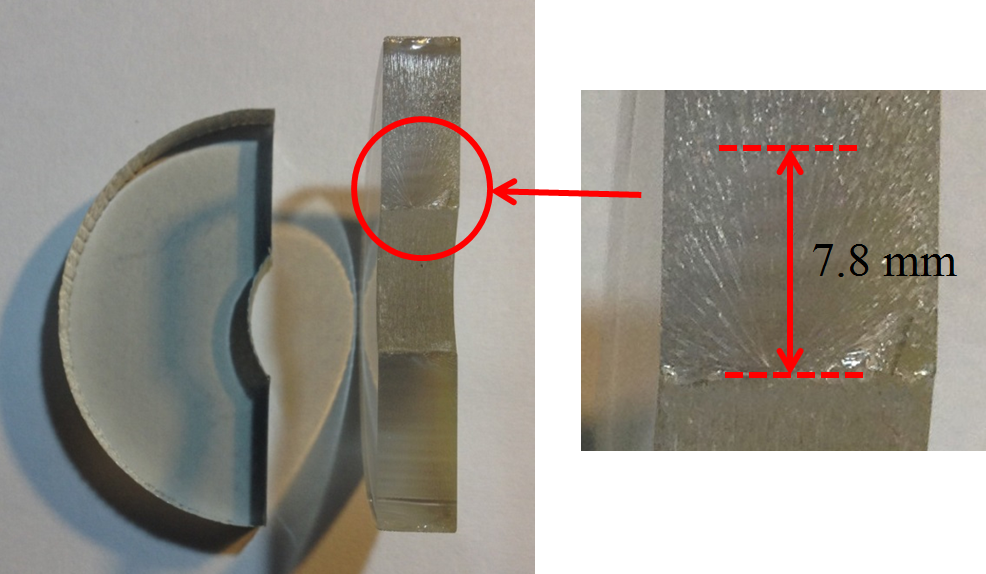


**Figure S5** Cracked sample; its critical crack area is a semicircle, and the diameter of the semicircle is equal to the thickness of the disc, i.e., 7.8 mm.

We initialize the details of the crack in the numerical simulation using the experimental critical crack measurements. As can be seen in Figure 5, the critical crack area of the cracked sample is a semicircle; the diameter of the semicircle is equal to the thickness of the disc, i.e., 7.8 mm. After inserting two semi-circular critical cracks, the variation in deviator stress (Figure 6) during crack propagation is computed using FEM in combination with LEFM.

(a)
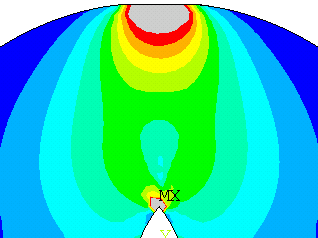
(b)
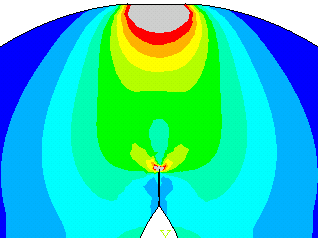
(c)
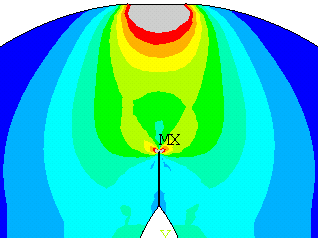

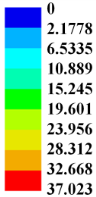


(d)
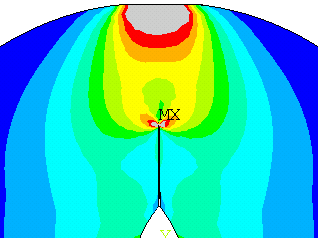
(e)
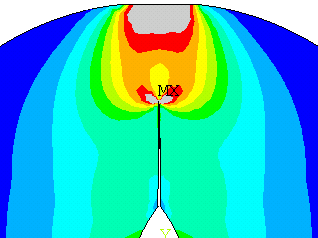
(f)
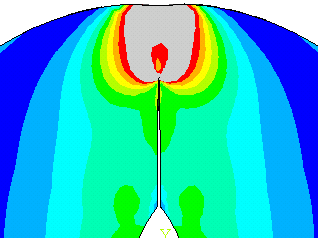


(g)
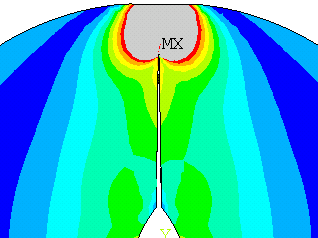
(h)
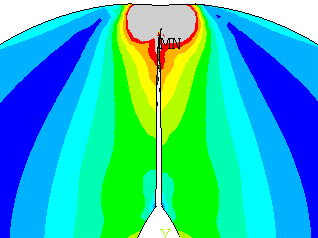
(i)
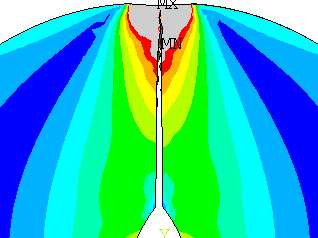


**Figure S6** Variation of the deviator stress (
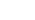
) during crack propagation in numerical simulation (unit: MPa).

**4 SIF analysis by photoelastic method**

For plane stress, the stress components can be expressed by the Westergaard stress function [6]:

$\begin{matrix} \sigma_{x}=\frac{K_{I}}{\sqrt{2\pi r}}\cos\frac{\theta}{2}(1-\sin\frac{\theta}{2}\sin\frac{3\theta}{2})+... \\ \sigma_{y}=\frac{K_{I}}{\sqrt{2\pi r}}\cos\frac{\theta}{2}(1+\sin\frac{\theta}{2}\sin\frac{3\theta}{2})+... \\ \tau_{xy}=\frac{K_{I}}{\sqrt{2\pi r}}\cos\frac{\theta}{2}\sin\frac{\theta}{2}\cos\frac{3\theta}{2}+... \end{matrix}$ (4)

where $x$ is the crack propagation direction, $y$ is the normal direction of the crack plane,
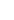
,
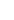
 and
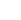
 are stresses in *x*-*y* plane, *K*_I_ is the stress intensity factor, and $r$ and $\theta$ are the polar coordinates (as shown in Figure 7). The relationship between polar coordinates and rectangular coordinates can be expressed as

$r=\sqrt{x^{2}+y^{2}},\theta=\arctan\frac{y}{x}$ (5)


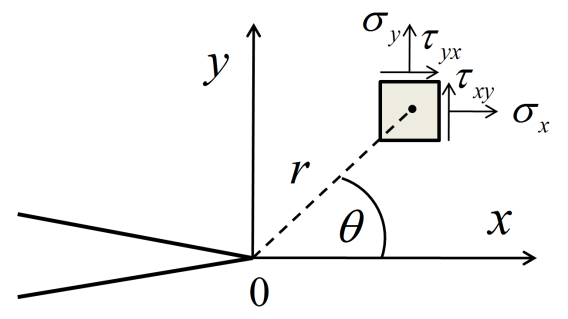


**Figure S7** Stress components around the crack tip in the stress plane.

Based on the stress-optical law ($\sigma_{1}^{'}-\sigma_{2}^{'}=\frac{n\cdot f_{0}}{t}$) and Equations (3), the relationship between the isochromatic fringe and plane stress can be expressed as

$\frac{n\cdot f_{0}}{t}=\sqrt{(\sigma_{x}-\sigma_{y})^{2}+4\tau_{xy}^{2}}$ (6)

where *n* is the fringe order, *f*_0_ is the stress fringe value and *t* is the thickness of the model.

Considering that the higher-order terms in Equation (4) are constants, (4) can be rewritten as:

$\begin{matrix} \sigma_{x}=\frac{K_{I}}{\sqrt{2\pi r}}\cos\frac{\theta}{2}(1-\sin\frac{\theta}{2}\sin\frac{3\theta}{2})+\sigma_{ox} \\ \sigma_{y}=\frac{K_{I}}{\sqrt{2\pi r}}\cos\frac{\theta}{2}(1+\sin\frac{\theta}{2}\sin\frac{3\theta}{2})+\sigma_{oy} \\ \tau_{xy}=\frac{K_{I}}{\sqrt{2\pi r}}\cos\frac{\theta}{2}\sin\frac{\theta}{2}\cos\frac{3\theta}{2}+\tau_{oxy} \end{matrix}$ (7)

Finally, the crack-tip SIF can be calculated by the isochromatic fringe order $n$ and its relevant polar coordinates $r$ and $\theta$(as shown in Figure 8):

$\begin{matrix} \sqrt{(\frac{-2K_{I}}{\sqrt{2\pi r}}\cos\frac{\theta}{2}\sin\frac{\theta}{2}\sin\frac{3\theta}{2}+\sigma_{ox}-\sigma_{oy})^{2}+4(\frac{K_{I}}{\sqrt{2\pi r}}\cos\frac{\theta}{2}\sin\frac{\theta}{2}\cos\frac{3\theta}{2}+\tau_{oxy})^{2}} \\ =\frac{n\cdot f_{0}}{t} \end{matrix}$ (8)


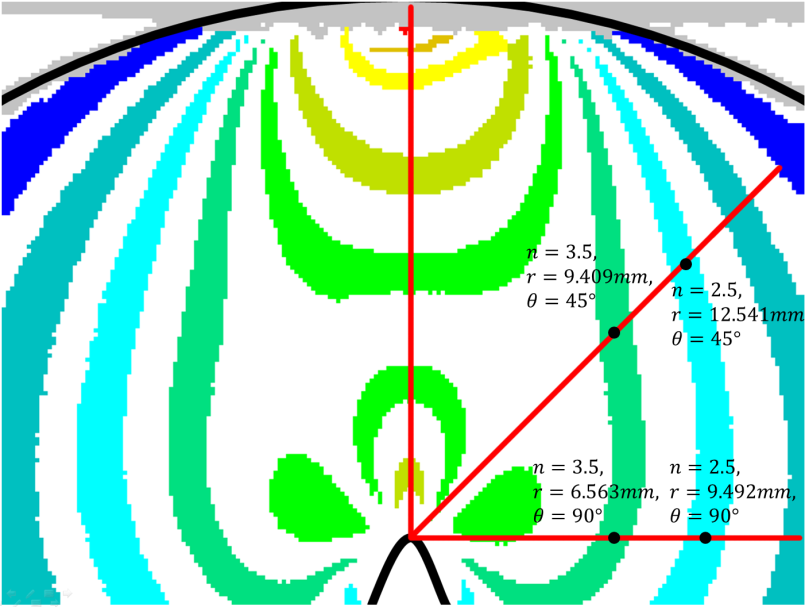

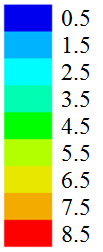


**Figure S8** Isochromatic fringe order $n$ (ranging from 0.5 to 8.5) and its relevant polar coordinates $r$ and $\theta$.

**5 Repetitive results (Sample 2)**

Figures S9 and S10 are the repetitive results of half-integer isochromatic fringes at the near-tip region during fracturing and deviator stress at half-integer isochromatic fringes over 80 µs. The crack tip velocity, stress field and their relationship in fracturing have been presented and discussed in main text.


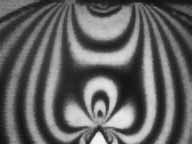

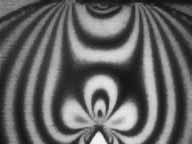

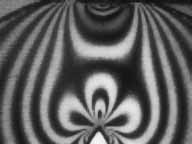


(a) (b) (c)


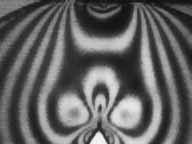

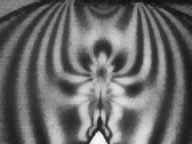

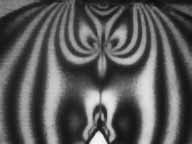


(d) (e) (f)


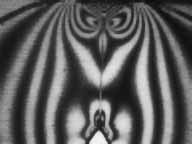

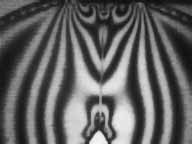

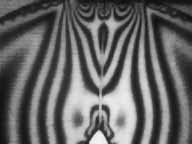


(g) (h) (i)

**Figure S9** Repetitive test: Half-integer isochromatic fringes at the near-tip region during crack propagation when the captured interval time is 10 µs. (a) 0 µs, (b) 10 µs, (c) 20 µs, (d) 30 µs, (e) 40 µs, (f) 50 µs, (g) 60 µs, (h) 70 µs, (i) 80 µs.


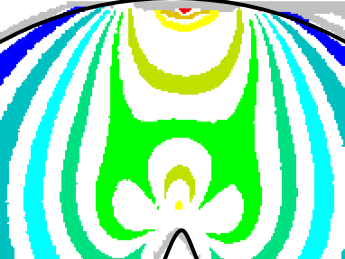

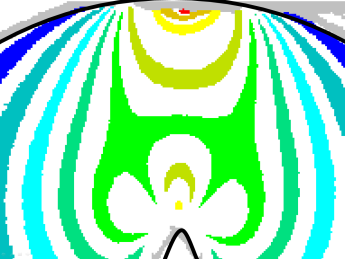

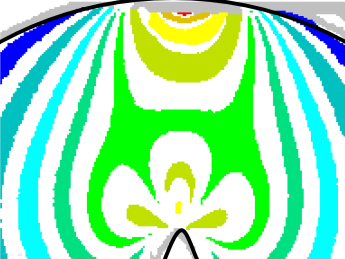

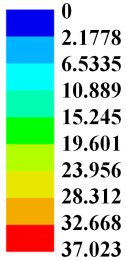


(a) (b) (c)


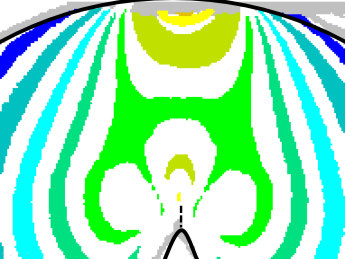

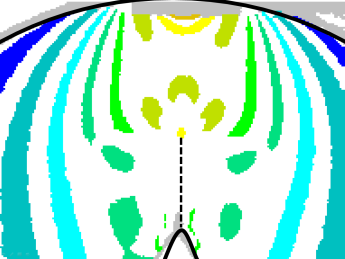

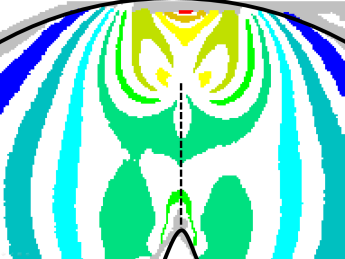


(d) (e) (f)


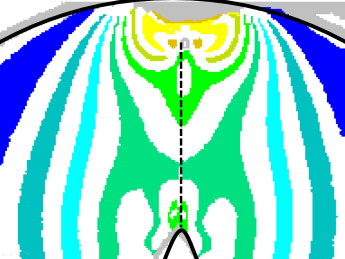

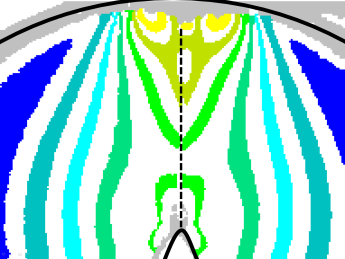

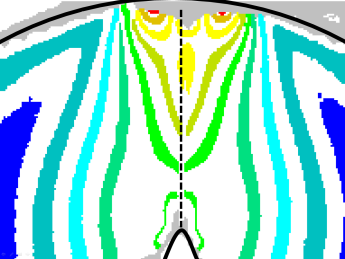


(g) (h) (i)

**Figure S10** Repetitive test: Deviator stress at half-integer isochromatic fringes. (a) 0 µs, (b) 10 µs, (c) 20 µs, (d) 30 µs, (e) 40 µs, (f) 50 µs, (g) 60 µs, (h) 70 µs, (i) 80 µs

**References**

1. Atkinson, B. K. (1987). Introduction to fracture mechanics and its geophysical applications - fracture mechanics of rock - 1. Fracture Mechanics of Rock, 1–26.
2. Sharon, E., & Fineberg, J. (1999). Confirming the continuum theory of dynamic brittle fracture for first cracks. Nature International Weekly Journal of Science, 397, 333-335.
3. Fowell, R. J. (1995). Suggested method for determining mode I fracture toughness using cracked chevron notched brazilian disc (ccnbd) specimens. International Journal of Rock Mechanics & Mining Sciences & Geomechanics Abstracts, 32(7), 57–64.
4. Ingraffea, A. R. (1987). Theory of crack initiation and propagation in rock - fracture mechanics of rock - 3. Fracture Mechanics of Rock, 71–110.
5. Ju, Y., Xie, H. P., Zheng, Z. M., Lu, J. B., Mao, L. T., Gao, F., & Peng, R. D. (2014). Visualization of the complex structure and stress field inside rock by means of 3D printing. China Science Bulletin, 59(36):5354-5365.
6. Hyde T. H., Warrior N. A. (1990) An improved method for the determination of photoelastic stress intensity factors using the Westergaard stress function. International Journal of Mechanical Sciences, 32(3):265-273.
